# Use of a confocal optical device for centring a diamond anvil cell in single-crystal X-ray diffraction experiments

**DOI:** 10.1107/S1600576724007829

**Published:** 2024-09-20

**Authors:** Chengdao Hu, Cameron J. G. Wilson, Daniel M. Scully, Tobias Stuerzer, Simon Parsons

**Affiliations:** a EastChem School of Chemistry and Centre for Science at Extreme Conditions The University of Edinburgh King’s Buildings, West Mains Road EdinburghEH9 3FJ United Kingdom; b Micro-Epsilon UK Ltd, No. 1 Shorelines Building, Shore Road, BirkenheadCH41 1AU, United Kingdom; c Bruker AXS GmbH, Oestliche Rheinbrueckenstrasse 49, 76187Karlsruhe, Germany; The University of Western Australia, Australia

**Keywords:** diamond anvil cells, high-pressure experiments, sample alignment

## Abstract

High-pressure crystallographic data can be measured using a diamond anvil cell (DAC), which allows the sample to be viewed only along a cell vector which runs perpendicular to the diamond anvils. Although centring a sample perpendicular to this direction is straightforward, methods for centring along this direction often rely on sample focusing, measurements of the direct beam or short data collections followed by refinement of the crystal offsets. These methods may be inaccurate, difficult to apply or slow. Described here is a method based on precise measurement of the offset in this direction using a confocal optical device, whereby the cell centre is located at the mid-point of two measurements of the distance between a light source and the external faces of the diamond anvils viewed along the forward and reverse directions of the cell vector. It is shown that the method enables a DAC to be centred to within a few micrometres reproducibly and quickly.

## Abstract 2

Centring a diamond anvil cell prior to X-ray diffraction measurements is facilitated by use of a confocal optical device.

## 1. Introduction

**Figure 1:**
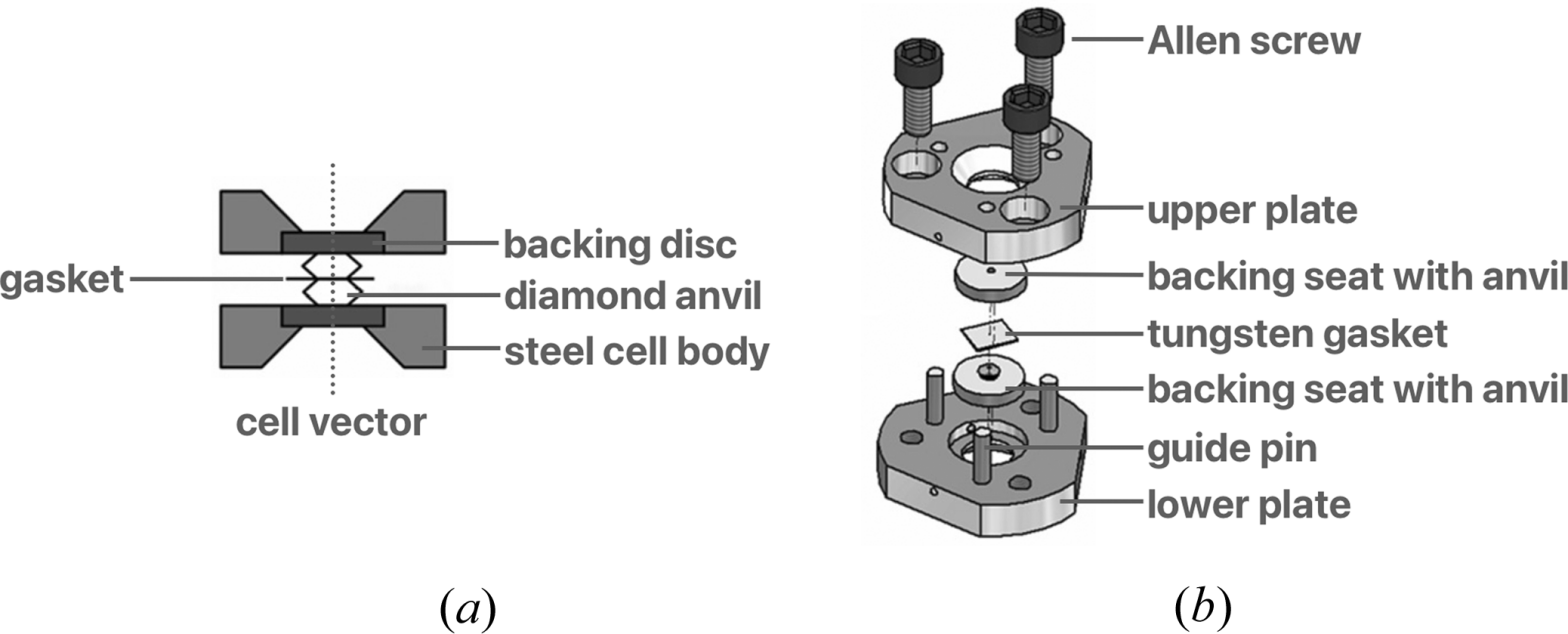
(*a*) A cross section and (*b*) components of a Merrill–Bassett DAC. Reproduced with permission from Moggach *et al.* (2008 ▸).

High-pressure single-crystal X-ray diffraction using diamond anvil cells (DACs) has become a popular area of crystallographic research, both at central facilities and in home laboratories. It has been applied to many areas of chemical crystallography, including pharmaceuticals and functional materials such as metal–organic frameworks (McKellar & Moggach, 2015 ▸; Moggach & Oswald, 2020 ▸). A DAC consists of two diamond anvils opposed across a small hole drilled into a metal gasket which acts as a sample chamber. The backing seats used to support the diamonds have conical holes to allow optical access for alignment as well as spectroscopic and diffraction measurements. The assembly is held in a clamp consisting of steel plates which support the backing discs and apply load. A sketch showing a cross section of the commonly used Merrill–Bassett DAC is provided in Fig. 1 ▸ (Merrill & Bassett, 1974 ▸; Moggach *et al.*, 2008 ▸).

Before diffraction data collection can commence, the DAC needs to be centred on the goniometer. In this work we present a method for centring a DAC which is particularly suited to home laboratories, using a confocal chromatic optical sensor for precise (<<1 µm) distance measurement. The polychromatic white light is focused onto the target surface by a multi-lens optical system. The special lens arrangement splits the white light into monochromatic wavelengths by controlled chromatic aberration. A specific distance is assigned to each wavelength by factory calibration. Only the wavelength which is exactly focused on the target is used for the measurement. An optical arrangement images the light reflected onto a light-sensitive sensor element. This sensor element detects the corresponding spectral colour to achieve measurement of distance (MicroEpsilon, 2024 ▸).

## 2. Experimental

### 2.1. Sample preparation

Crystals of glyphosate (Sigma–Aldrich) were grown by slow evaporation of an aqueous solution of concentration 10 mg ml^−1^ (Wilson *et al.*, 2023 ▸). The crystals are monoclinic with a plate-like morphology developed in (100).

A crystal was loaded into a Merrill–Bassett DAC (Merrill & Bassett, 1974 ▸) with a half opening angle of 38°, 600 µm Boehler–Almax cut diamonds and conically ground tungsten carbide backing plates (Moggach *et al.*, 2008 ▸). A tungsten gasket of initial thickness 300 µm was indented to a thickness of approximately 98 µm, and a hole of diameter 300 µm was drilled by spark erosion. A 4:1 mixture of methanol and ethanol was used as the pressure-transmitting medium. The sample pressure was determined to be 2.2 GPa using ruby fluorescence (Mao *et al.*, 1986 ▸; Shen *et al.*, 2020 ▸).

Single-crystal X-ray diffraction data were collected on a Bruker AXS D8 Venture three-circle (2θ, ω and φ with χ fixed at 54.74°) diffractometer incorporating an Incoatec Mo *K*α (λ = 0.71073 Å) microsource.

**Figure 2:**
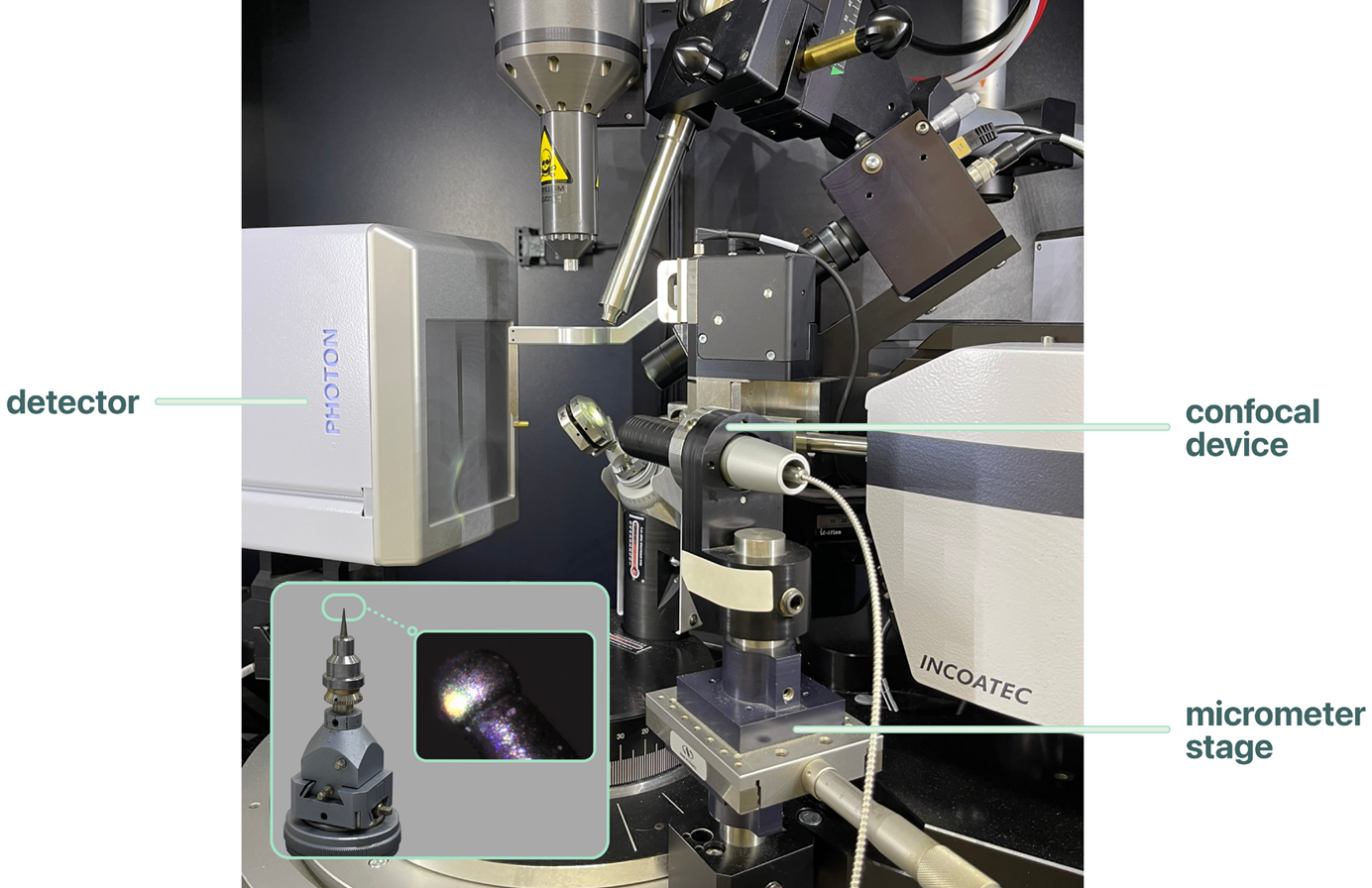
A diffractometer configuration showing the confocal device mounted on the diffractometer stage. The inset shows a steel ball mounted on a goniometer head as used for the sensor alignment.

A MicroEpsilon IFS2406-3 confocal device was fitted to the base of the goniometer and operated with a confocal DT2421 control unit (Fig. 2 ▸). The static resolution of the sensor is 32 nm. This is well beyond the fluctuations resulting from vibrations in the goniometer stage, which are of the order of 1 µm. The sensor was set to measure surfaces in ‘standard shiny’ mode with a measuring rate of 0.1 kHz. Other specification data are available in a technical note (MicroEpsilon, 2024 ▸).

### 2.2. Centring procedure

The diffractometer is equipped with a video camera mounted on a micrometer stage for viewing and centring the sample; the micrometer stage allows the camera focus to be adjusted. A sample contained in a DAC may only be viewed directly along the cell vector [Fig. 1 ▸(*a*)], defined by the line passing through both diamonds perpendicular to the culet faces. The DAC is mounted on a standard Huber 1004 goniometer head so that the cell vector is parallel to one of the centring adjustment directions. A goniometer centring position is selected so that the cell axis lies along the viewing direction of the video camera.

Centring of the cell in the directions perpendicular to the cell vector is accomplished optically, in the same way as for a crystal on a fibre or loop, by ensuring that the centre of the crystal remains at the same absolute position in the video microscope image after rotating it by 180° about the φ axis. The success of this procedure depends on the diamond alignment, as the high refractive index of diamond means that even a small misalignment can displace the image (*e.g.* a 2° misalignment displaces the image by 30 µm for a diamond thickness of 1.6 mm) (Angel *et al.*, 2000 ▸).

Initial centring of the crystal along the cell vector can also be performed using the video camera. An initial reading is taken on the video camera stage micrometer, and the camera focus is then adjusted so that the sample is in focus. A second micrometer reading is taken and the video camera is moved back to the average of the two micrometer readings. The image focus is then re-established using the goniometer head adjustor screw parallel to the viewing direction of the video camera. The cell can be rotated by 180° in φ to check that the sample remains in focus; if not, the procedure can be iterated until the sample is in focus when viewed along both forward and reverse directions along the cell vector. The success of this method relies on both diamonds having the same thickness.

**Figure 3:**
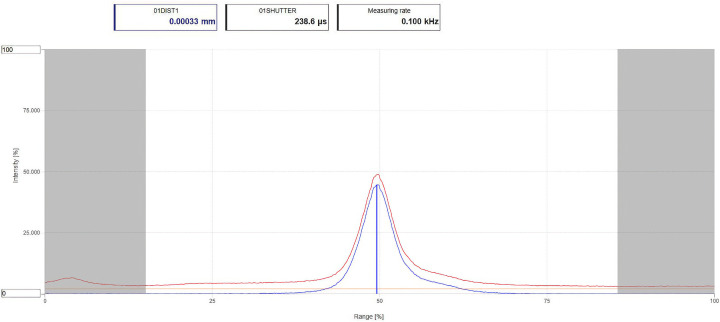
The user interface for the confocal device. The red and blue traces refer to the raw and optically corrected readings, respectively.

More accurate centring along the cell vector can be accomplished with the confocal device. The cell is rotated using goniometer setting angles that place the cell vector along the beam path of the confocal device (Section 2.3). The device incorporates a light source which is projected onto the object, enabling measurement of the source–object distance within a range of 3 mm. The control interface is shown in Fig. 3 ▸. First, the distance of the confocal device is set so that its readout on the interface is in the centre of its range. This distance of the readout is set to a reference value of zero and the cell is then rotated in φ by 180°. The new distance readout is noted and the position of the cell then adjusted using the goniometer head so that the distance is halved in value. The distance reading is reset to zero and the cell is rotated through 180° in φ, *i.e.* back to its original position. The procedure can be iterated if necessary, so that the distance readout is zero to within ±3 µm when the cell is viewed along both the forward and reverse directions along the cell vector.

### 2.3. Alignment of the sensor

The position of the sensor on the goniometer defines the value of ω to which the DAC should be moved for use of the confocal device. This value can be determined by mounting, indexing and face-indexing a crystal with well developed faces, such as NaCl. A face can then be oriented in a vertical position and the crystal rotated in ω until the signal from the confocal device is maximized. The mount for the device should enable small alignment adjustments to be made to the orientation of the device, also aiming to maximize the signal, ensuring that there is a single peak which has a symmetrical Gaussian peak shape across the range of distance adjustment. The latter is a particularly sensitive criterion, but fine adjustments can also be made with direct visual feedback on the position of the sensor by replacing the alignment crystal with a steel ball (diameter 300 µm, Fig. 2 ▸ inset), commonly used for diffractometer beam alignment. The ball can be accurately centred optically and then used as a reference for aligning the sensor in its mount.

### 2.4. Validation of centring using diffraction

The centring can be validated by carrying out a short data collection using different combinations of ω and 2θ settings from either side of the direct beam. The strategy used, which can take as little as three minutes to run for a well diffracting sample, is shown in Table 1 ▸. This diffraction centring strategy was developed for use on the XIPHOS facility, where a sample or even a DAC can be enclosed in a cryostat (Probert *et al.*, 2010 ▸). Reflections are harvested from the first two runs of the strategy in Table 1 ▸ and used for indexing the diffraction pattern of the sample. The unit-cell and instrument model parameters, but not the sample offsets, are refined using these data, which should consist of at least 200 reflections. Reflections from the third run are then harvested and used (together with data from the first two runs) to refine only the crystal offsets, holding other parameters fixed. For our work using laboratory sources with beam diameters of 90–130 µm and crystal dimensions of *ca* 100 µm, offsets within 10 µm of zero, which are commensurate with typical standard uncertainties obtained from diffraction centring, are considered adequate.

### 2.5. Data collections

Diffraction data were collected using the strategy shown in Table 2 ▸, which is based on that described by Dawson *et al.* (2004 ▸) but with runs split so that shading is minimized at the beginning of each run. Data were collected with the cell in its centred position and in positions deliberately displaced by ±30 and ±60 µm along the cell vector. They were reduced using the *APEX4* software suite (Bruker, 2021 ▸), ensuring that regions shaded by the body of the DAC were omitted during integration. A correction for absorption by the sample and cell, shading by the gasket, and other systematic errors was carried out using the multi-scan procedure in *SADABS* (Krause *et al.*, 2015 ▸). Crystal structures were refined using the *OLEX-2* interface to *SHELXL* (Dolomanov *et al.*, 2009 ▸; Sheldrick, 2015 ▸).

## 3. Results and discussion

### 3.1. Centring procedure using the optical sensor

Although diamonds are optically transparent, it is sometimes not possible to obtain a clear view of a sample from both sides of a DAC. This may be because a crystal has been grown *in situ* and the crystalline region of the sample is obscured; a sample may have fragmented; the sensitivity of a sample may mean that it needed to be loaded quickly with contaminated mother liquor as a pressure-transmitting medium; the medium may partially dissolve the sample and become coloured; optical effects in partially vitrified media may also occur; or, sadly, the outer faces of the diamonds may be dirty. In short, there are many reasons why a clear view of a sample might not be obtained, which can make methods of centring based on focusing the sample image from two opposite directions difficult to apply. Moreover, even when a clear image can be obtained, assessment of whether an image is focused or not can be somewhat subjective and dependent on the quality of the lighting and optics on the viewing device being used. Our aim in incorporating the optical sensor into the centring procedure for a DAC was to replace focus-based centring methods with one which is both based on numerical measurements and less sensitive to the characteristics of the sample. Although the method has been applied to DACs, it could in principle be used for any experiment where the view of the sample is restricted, for example when a sample is surrounded by other material, such as can occur in a capillary.

Whatever method of centring is used, it is prudent to validate the position of the sample using diffraction by carrying out a short data collection and refining the sample offsets. We have found in testing (see below) that the diffraction and optical centring were usually found to coincide within 10 µm. This is within tolerance for most in-house experimental work with a beam size of ∼100 µm and typical crystal dimensions of 0.05–0.2 mm (see also Section 2.4). We expect that the procedure described will be less useful on synchrotron beamlines, where beam and sample sizes are typically an order of magnitude smaller. In any case, procedures based on beam scanning using precisely motorized sample stages at these facilities already provide highly efficient methods for sample location and centring.

**Figure 4:**
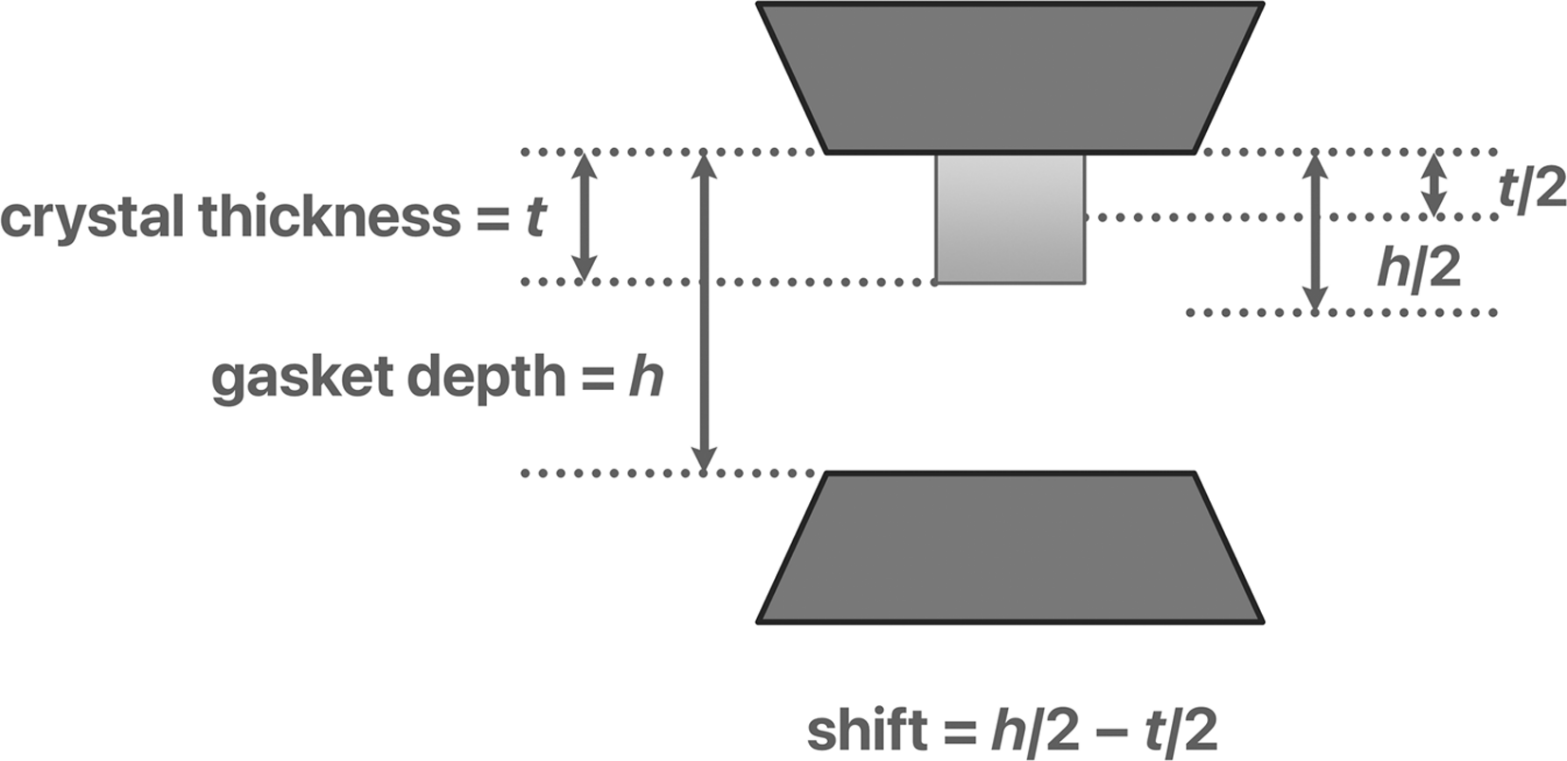
The shift required between the mid-point of the diamond culets (dark grey) and the centre of the crystal (light grey) is (*h* − *t*)/2, where *h* is the gasket depth (typically measured during indenting) and *t* the thickness of the crystal.

Should further adjustments of the sample be necessary after the diffraction measurements, these can be readily applied with the aid of the confocal sensor. This can occur because the procedure outlined above finds the position of the mid-point between the diamond anvils rather than the centre of the sample, which is typically not located at the exact geometric centre of the gasket but mounted to one of the two faces of the anvil diamonds. For plate-like crystals in particular, these positions may differ by several tens of micrometres. If the gasket height (*h*) and thickness of the crystal (*t*) are known, the offset between the centre of the anvils and the centre of the crystal is (*h* − *t*)/2 (Fig. 4 ▸). This further adjustment can be applied after the centring described in Section 2.2 and before diffraction centring to position the crystal with higher accuracy.

Although in-house high-pressure work is usually still carried out using conventional manual goniometer heads, motorized heads are becoming much more common for ambient-pressure measurements. Very convenient procedures are available that allow a user to select the centre of a sample with a mouse click or even rely on image-recognition algorithms to identify the sample. Application of this approach to high-pressure work would be very attractive because the precision of adjustments on motorized heads is finer than that on manual heads, provided the weight of the cell can be accommodated. Use of a motorized goniometer head would be immediately applicable to centring perpendicular to the cell vector. The numerical feedback provided by the confocal centring procedure described here would also provide the distance adjustments required for centring along the cell vector, introducing the potential for essentially automated DAC centring.

### 3.2. Data collection tests

In order to illustrate the importance of centring, the results of data collections in which a sample was displaced by 30 µm and then 60 µm along positive and negative directions along the cell vector were compared with those obtained after accurate centring. The crystal and refinement data from these tests are listed in Table 3 ▸.

**Figure 5:**
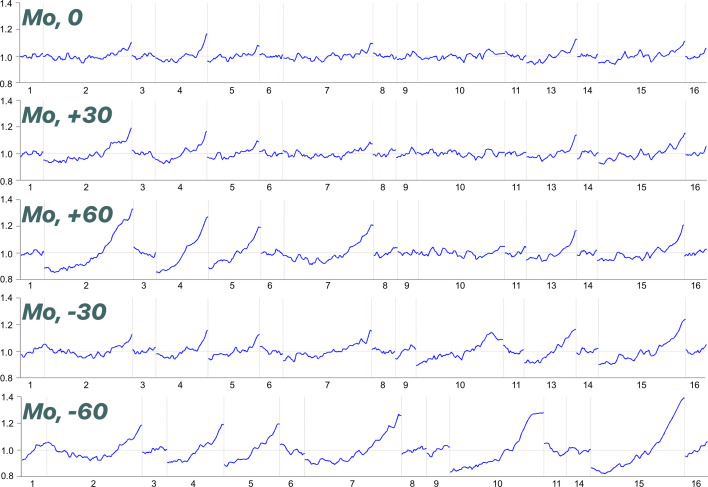
Scale variation graphs for Mo X-ray radiation measurement with different *y* offsets applied.

The refined offsets obtained after diffraction centring agree within error with the offsets measured with the confocal device. However, the unit-cell volumes calculated after each of the data collections in Table 3 ▸ span a range of 0.45 Å^3^, which is high by comparison with the standard uncertainties quoted (typically 0.11 Å^3^). Acceptable refinement statistics were obtained for all data sets despite the applied offsets, though *R*_1_ does experience a modest increase at higher offsets. The scale factors applied by the multi-scan correction for systematic errors show increasing variation for larger offsets (Fig. 5 ▸). Nevertheless, the insensitivity of the refinement statistics to the applied offsets attests to the success of the correction in this case.

## Figures and Tables

**Table 1:** Strategy for short data collections used for validation of centring The step size is usually set to 0.5° and the time per step is typically 1–5 s. The detector distance is 63 mm, 2θ = 0° and χ = 54.74° in all runs.

Run	Scan angle (°)	Fixed angle (°)
1	13.00 to −17.00 in ω	φ = 270.00
2	13.00 to −17.00 in ω	φ = 90.00
3	65.00 to 115.00 in φ	ω = 0.00

**Table 2:** Strategy for diffraction data collections The step size is usually set to 0.3° and the time per step is typically 5–60 s. The detector distance is 63 mm and χ = 54.74° in all runs.

Run	2θ (°)	φ (°)	ω range (°)
1	11.00	270.00	7.00 to 22.00
2	11.00	270.00	15.00 to −45.00
3	−11.00	270.00	345.00 to 360.00
4	−11.00	270.00	353.00 to 320.00
5	11.00	270.00	187.00 to 220.00
6	11.00	270.00	195.00 to 180.00
7	−11.00	270.00	165.00 to 225.00
8	−11.00	270.00	173.00 to 158.00
9	11.00	90.00	7.00 to 22.00
10	11.00	90.00	15.00 to −45.00
11	−11.00	90.00	345.00 to 360.00
12	−11.00	90.00	353.00 to 320.00
13	11.00	90.00	187.00 to 220.00
14	11.00	90.00	195.00 to 180.00
15	−11.00	90.00	165.00 to 225.00
16	−11.00	90.00	173.00 to 158.00

**Table:** The Bruker coordinate system places **x** along the X-ray beam from source to sample, **z** vertical and pointing up, and **y** making a right-handed set. The DAC was mounted so that the cell vector would lie along **y** if all the setting angles were at zero, so that the values of the *y* offset in this table correspond to displacements along the cell vector.

Empirical formula: C_3_H_8_NO_5_P	Crystal system: Monoclinic	Space group: *P*2_1_/*c*	Resolution limit: 0.7 Å

**Table:** 

		*y* offset				
		0 (centred)	30	60	−30	−60
Unit cell	*a* (Å)	8.6274 (12)	8.6261 (13)	8.6232 (11)	8.6262 (12)	8.6274 (13)
	*b* (Å)	7.7307 (5)	7.7305 (6)	7.7303 (5)	7.7299 (5)	7.7301 (6)
	*c* (Å)	9.4613 (7)	9.4604 (7)	9.4606 (6)	9.4620 (7)	9.4631 (7)
	α (°)	90.00	90.00	90.00	90.00	90.00
	β (°)	109.406 (8)	109.414 (8)	109.414 (7)	109.417 (8)	109.407 (9)
	γ (°)	90.00	90.00	90.00	90.00	90.00
	*V* (Å^3^)	595.18 (11)	594.99 (11)	594.79 (10)	595.04 (11)	595.24 (11)

Domain translation	*x* (mm)	−0.004 (5)	−0.004 (5)	−0.005 (5)	−0.013 (5)	−0.009 (5)
	*y* (mm)	0.004 (10)	0.027 (9)	0.051 (10)	−0.0045 (11)	−0.053 (9)
	*z* (mm)	0.000 (5)	0.002 (5)	−0.004 (5)	−0.004 (5)	0.000 (5)

*R*_1_ (%)		2.56	2.65	2.85	2.78	2.60
*wR*_2_ (%)		5.44	5.39	6.41	6.48	6.42
*R*_int_ (%)		4.17	4.37	4.34	4.56	4.30
Total No. of reflections		2693	2772	2672	2724	2520
No. of unique reflections		339	356	352	362	362
Reflections with *I* ≥ 2σ(*I*)		296	299	300	292	293
Completeness (%)		27.0	26.8	26.7	26.9	25.7
Average *I*/σ(*I*)		27.70	27.49	26.98	26.95	24.61
